# Heart valve operations associated with reduced risk of death from mitral valve disease but other operations associated with increased risk of death: a national population-based case–control study

**DOI:** 10.1186/s13019-019-0984-x

**Published:** 2019-09-14

**Authors:** Ruo-Ling Li, Ci-Wen Luo, Yung-Chyuan Ho, Shiuan-Shinn Lee, Yu-Hsiang Kuan

**Affiliations:** 1 Department of Public Health, Institute of Public Health, Chung Shan Medical University, Taichung, Taiwan; 2 Department of Medical Management, Division of Thoracic Surgery, Chung Shan Medical University Hospital, Taichung, Taiwan, Republic of China; 3 Department of Pharmacology, School of Medicine, Chung Shan Medical University, No.110, Sec. 1, Jianguo N. Rd, Taichung, Taiwan, Republic of China; 4 Department of Pharmacy, Chung Shan Medical University Hospital, Taichung, Taiwan; 5 School of Medical Applied Chemistry, Chung Shan Medical University, Taichung, Taiwan

**Keywords:** Heart valve operations, Mitral valve disease, Other operations, Risk of death

## Abstract

**Background:**

Mitral valve disease is the most common heart valve disease worldwide. Heart valve operation is the predominant treatment strategy for heart valve disease. This study analyzed the death risk from heart valve disease with respect to the frequency of heart valve operation and other operations in patients with mitral valve disease.

**Materials and methods:**

We conducted a retrospective nationwide population-based case–control study using a claims dataset from Taiwan’s National Health Insurance Research Database. The case and control groups enrolled mitral valve disease patients from 2002 to 2013 who had either underwent an heart valve operation procedure or not, respectively. Conditional logistic regression was estimated the odds ratios (ORs) associated with various risk factors for heart valve operation-related death, including other operations and comorbidities.

**Results:**

A total of 25,964 patients with mitral valve disease were recruited for the study and divided into heart valve operation (600 patients) and non-heart valve operation (25,364 patients) groups. After matching, a total of 1800 non-heart valve operation patients were selected for final analysis. Heart valve operation was associated with decreased risk of death (adjusted OR [aOR] 0.439), but operations related to other cardiovascular disease (CVD, aOR 3.691), respiratory conditions (aOR 3.210), and the urinary system (aOR 1.925) were associated with increased risk of death for patients with mitral valve disease. Patients with mitral valve disease and diabetes (aOR 1.505), chronic kidney disease (CKD, aOR 3.760), or emphysema (aOR 2.623) also had a higher risk of death. Patients who underwent more heart valve operations had a lower risk of death from mitral valve disease, but patients who underwent more other operations had a higher risk of death from mitral valve disease.

**Conclusions:**

The death risk for patients with mitral valve disease patients could be lowered by more frequently performing heart valve operations. However, the risk of death is increased for patients with mitral valve disease who more frequently undergo other operations, chiefly those for other CVD system, respiratory conditions, and urinary system, or have comorbidities such as diabetes, chronic kidney disease, and emphysema.

## Background

Heart valve disease, defined as a valve dysfunction induced by abnormal structure or function of the aortic or mitral valve in the heart, is a common cardiovascular disease worldwide [1]. The prevalence of heart valve disease by age ranges from 0.7% for 18 to 44-year olds to 11.7% for those aged 75 years or older. In the United States, the prevalence of heart valve disease is estimated to be 2.5%. Mitral valve disease, especially mitral regurgitation, is the most common heart valve disease. The prevalence of mitral valve disease is estimated to be 1.8% in the United States population [2]; 664,369 patients died from heart valve disease from 1979 to 2009 in the United States, and the mortality rate increased 2.8% per year in the 1979 to 2009 time period. The percentage of deaths attributable to various heart valve diseases was as follows: 45.2% from nonrheumatic aortic valve disease, 9.8% from nonrheumatic mitral valve disease, 20.1% from rheumatic heart disease, and 20.5% from endocarditis [3].

The predominant treatment strategy for moderate to severe heart valve disease is a heart valve operation, which constitutes 10 to 20% of all cardiac operations in the United States [4, 5]. A study reviewed the Nationwide Inpatient Sample database from 2003 to 2012 and determined that the mortality and morbidity of patients undergoing triple valve surgery are higher than those of the overall in-hospital patient population [6]. Patients undergoing a redo mitral valve operation had higher comorbidities and operative mortalities. After risk-factor adjustment, the expected rate of mortality for patients undergoing redo mitral valve operations was higher than that for patients undergoing a primary mitral valve operation from 2002 to 2009. However, the observed–expected ratio of mortality for redo-mitral valve operations was lower than for primary mitral valve operations from 2010 to 2014 [7]. The aim of this study was to explore the death risk of heart valve diseases and the frequency with which patients with mitral valve diseases underwent other operations.

## Methods

### Data sources

Since 1995, Taiwan has administered its National Health Insurance (NHI) program that covers 99% of the Taiwanese population. The Longitudinal Health Insurance Database (LHID) 2010 contains complete original claims data of 1,000,000 beneficiaries enrolled in 2010, which were randomly sampled from the 2010 Registry for Beneficiaries of the National Health Insurance Research Database (NHIRD). The data set includes key characteristics of the beneficiaries, chiefly demographic data, treatments undergone, medications prescribed, and diseases diagnosed. Disease diagnoses are based on the International Classification of Diseases, Ninth Revision, Clinical Modifications (ICD-9-CM). This study was approved by the Institutional Review Board of the Chung Shan Medical University Hospital in Taichung, Taiwan.

### Identification of case and control

**Fig. 1:**
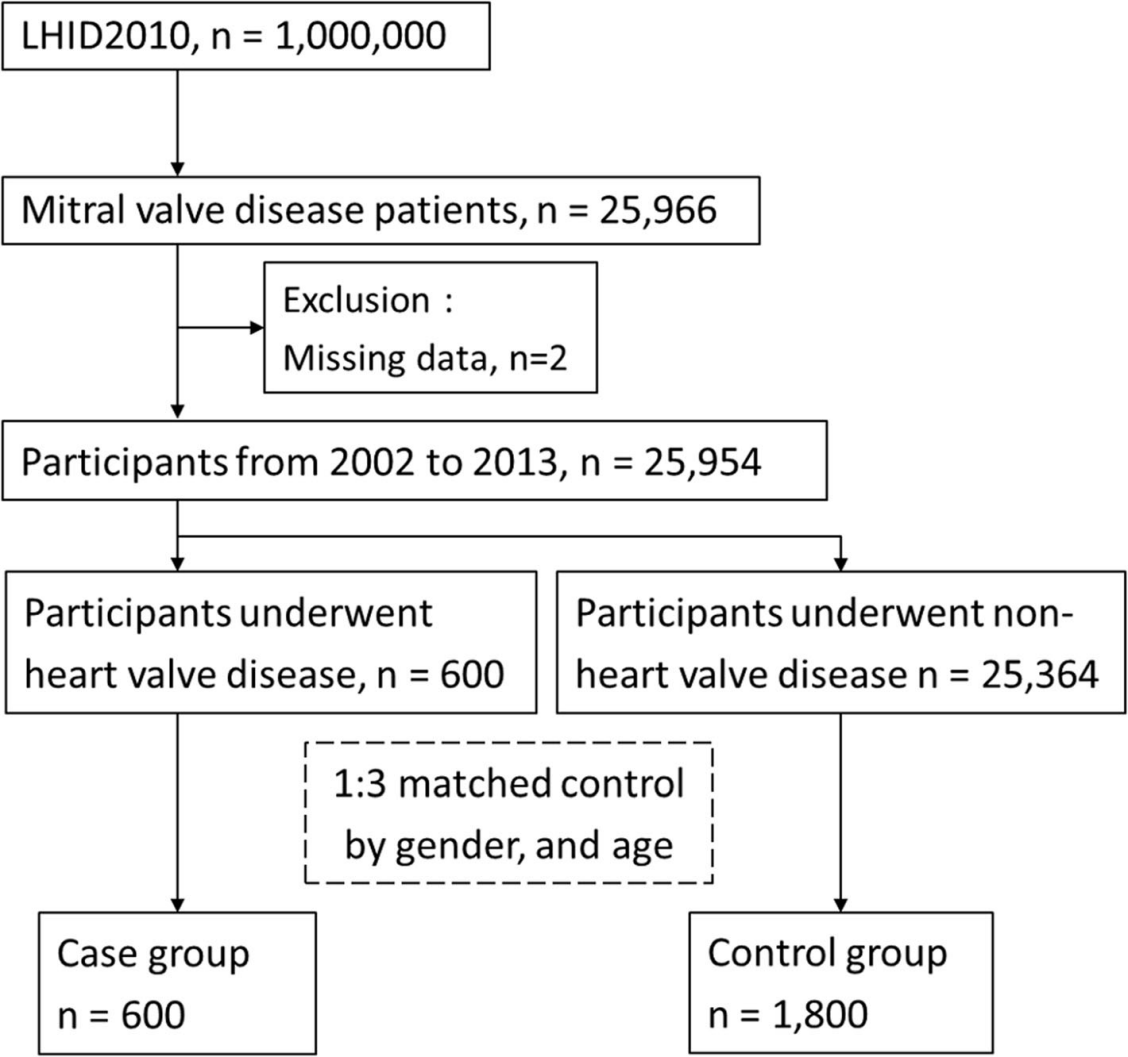
Flowchart of patient selection

Patients with mitral valve diseases were enrolled based on ICD-9-CM codes (093.21, 394, 396, 424, 746.5, 746.6) and whether they had visited the outpatient department more than twice or been hospitalized at least once because of pulmonary and cardiac diseases as documented in the LHID 2010 database (*n* = 25,996), excluding missing data (*n* = 2). The case group included 600 patients who had underwent the heart valve operations, as determined by ICD-9-OP code, ICD-9-CM codes (35.0, 35.1, 35.2), and more than one hospitalization. The other participants with mitral valve diseases but without the heart valve operations were matched 1:3 in age and sex with the control group (Fig. 1).

### Other operations and comorbidities

We analyzed other operations patients underwent during the study period, namely cardiovascular disease (CVD) system operations (based on ICD-9-OP code, ICD-9-CM codes: 35.3, 35.4, 35.5, 35.6, 35.7, 35.8, 35.9, 36.x. 37.x, 38.x, 39.x), nervous system operations (based on ICD-9-OP code, ICD-9-CM codes: 01.x. 02.x, 03.x, 04.x, 05.x), digestive system operations (based on ICD-9-OP code, ICD-9-CM codes: 42.x. 43.x, 44.x, 45.x, 46.x, 47.x, 48.x, 49.x, 50.x, 51.x, 52.x, 53.x, 54.x), respiratory condition operations (based on ICD-9-OP code, ICD-9-CM codes: 30.x. 31.x, 32.x, 33.x, 34.x, 35.x), urinary tract operations (based on ICD-9-OP code, ICD-9-CM codes: 55.x. 56.x, 57.x, 58.x, 59.x), and hemodialysis (based on ICD-9-OP code, ICD-9-CM codes: 35.95). Comorbidities were also analyzed, namely hypertension (ICD-9-CM codes: 401.x–405.x, 437.2), hyperlipidemia (ICD-9-CM codes: 272.x, A189), diabetes (ICD-9-CM codes: 250.x, A181), chronic obstructive pulmonary disease (ICD-9-CM codes: 490.x–496.x), emphysema (ICD-9-CM code: 492.x), heart failure (ICD-9-CM code: 428.x), cardiac dysrhythmia (ICD-9-CM code: 427.x), anxiety (ICD-9-CM code: 300.x), chronic kidney disease (CKD; ICD-9-CM code: 585.x), sleep disturbances (ICD-9-CM code: 780.5x), and anemia (ICD-9-CM code: 280.x) as documented in the Registry for Catastrophic Illness Patients database in the NHIRD; death was likewise analyzed.

### Statistical analysis

Conditional logistic regression was used to estimate the non-matched control and matched control odds ratios (ORs) and 95% confidence interval for the risk factors of heart valve operation. Potential risk factors were sex, age, low income, other operations, and comorbidities. Statistical analyses were performed using SAS 9.3 software, and *p* < 0.05 was considered statistically significant.

## Results

### Characteristics of the study population

In all, 25,964 patients were enrolled for the study (Table 1). A total of 600 (2.31%) patients had underwent heart valve operation, of which 322 (53.7%) were female and had an average age of 62 (±16.17) years. After matching, a total of 1800 non-heart valve operation patients were selected for final analysis. Baseline demographics such as sex, age, and income level of the case and control groups were not significantly different. Except nervous system operations, the prevalence of operations underwent by the two groups was significantly different, especially CVD operations (92.5%), which were more frequently performed in patients who underwent a heart valve operation. Except for emphysema, comorbidities of the two groups were not significantly different with respect to heart valve operation. Patients with emphysema (1.89%) underwent fewer heart valve operations.

**Table 1:** Basic characteristics of the study participants from 2002 to 2013

	Case (*n* = 600)	Control			
		Non-Matched (n = 25,364)	*P*	Matched (*n* = 1800)	*P*
Gender					
Female	322 (53.67%)	15,508 (61.14%)	0.2051	966 (53.67%)	1.0000
Male	278 (46.33%)	9256 (36.49%)		834 (46.33%)	
Age in 2008, Mean ± SD	62.37 ± 16.17	56.98 ± 19.26	<.0001	62.51 ± 16.13	0.9376
Low income					
Yes	349 (58.17%)	15,727 (62.01%)	0.5571	1022 (56.78%)	0.9208
No	251 (41.83%)	9637 (37.99%)		778 (43.22%)	
Other operation					
Other CVD system operation	555 (92.5%)	3365 (13.27%)	<.0001	341 (18.94%)	<.0001
Nervous system operation	24 (4.00%)	813 (3.21%)	0.0002	75 (4.17%)	0.5781
Digestives system operation	208 (34.67%)	4382 (17.28%)	<.0001	363 (20.17%)	<.0001
Breath system operation	118 (19.67%)	1070 (4.22%)	<.0001	91 (5.06%)	<.0001
Urinary system operation	37 (6.17%)	1119 (4.41%)	<.0001	91 (5.06%)	0.0041
Hemodialysis	22 (3.67%)	455 (1.79%)	<.0001	48 (2.67%)	0.2074
Comorbidity					
Hypertension	408 (68.00%)	14,430 (56.89%)	0.0513	1237 (68.72%)	0.8323
Hyperlipidemia	221 (36.83%)	10,013 (39.48%)	0.6667	834 (46.33%)	0.3355
Diabetes	198 (33.00%)	7498 (29.56%)	0.2915	649 (36.06%)	0.5501
COPD	225 (37.5%)	8205 (32.35%)	0.2259	674 (37.44%)	0.9707
Emphysema	7 (1.17%)	380 (1.50%)	<.0001	34 (1.89%)	<.0001
Heart failure	413 (68.83%)	5496 (21.67%)	<.0001	469 (26.06%)	0.0983
Cardiac dysrhythmias	372 (62%)	12,586 (49.62%)	0.3405	903 (50.17%)	0.3915
Anxiety	241 (40.17%)	14,800 (58.35%)	0.8703	1044 (58.00%)	0.8615
CKD	69 (11.5%)	2255 (8.89%)	<.0001	217 (12.06%)	0.5600
Sleep disturbances	280 (46.67%)	14,570 (57.44%)	0.7280	1037 (57.61%)	0.7544
Anemia	102 (17.0%)	4370 (17.23%)	0.7157	259 (14.39%)	0.1213

Abbreviation: *SD* Standard deviation, *CVD* Cardiovascular disease, *COPD* Chronic obstructive pulmonary disease, *CKD* Chronic kidney disease

### Associated diseases that resulted in death for patients who underwent heart valve operation

To evaluate the association between other operations and comorbidities in patients who underwent a heart valve operation and controls, multivariate logistic regression analysis was performed. Table 2 presents the adjusted OR (aOR) related to the factors of sex, age, other operations, and comorbidities. Heart valve operation was associated with decreased risk of death in matched or non-matched patients with mitral valve disease. With respect to other operations, other CVD, respiratory condition, urinary system operations, and hemodialysis were associated with a higher risk of death in matched or non-matched patients with mitral valve disease. Patients with CKD and emphysema had a higher risk for death in matched or non-matched patients with mitral valve disease.

**Table 2:** Logistic regression of heart valve operation and death in patients with mitral valve disease

	Non-matched	Matched
	aOR 95% CI	aOR 95% CI
Heart valve operation (reference: non-heart valve operation)		
Yes	0.625 (0.435–0.897)^*^	0.568 (0.341–0.946)^*^
Other operation (reference: without)		
Other CVD system operation	1.759 (1.492–2.075)^***^	2.419 (1.484–3.941)^***^
Nervous system operation	1.248 (0.937–1.662)	0.941 (0.395–2.244)
Digestives system operation	1.715 (1.484–1.982)^***^	1.222 (0.808–1.849)
Breath system operation	3.547 (2.959–4.251)^***^	2.753 (1.694–4.473)^***^
Urinary system operation	1.155 (0.911–1.466)	1.915 (1.028–3.568)^*^
Hemodialysis	5.210 (3.983–6.815)^***^	7.205 (3.728–13.92)^***^
Comorbidity (reference: without)		
Hypertension	1.141 (0.920–1.415)	0.628 (0.366–1.078)
Hyperlipidemia	0.634 (0.549–0.733)^***^	0.754 (0.502–1.132)
Diabetes	1.234 (1.069–1.424)^**^	1.547 (1.031–2.321)^*^
COPD	0.989 (0.855–1.143)	1.085 (0.720–1.634)
Emphysema	1.575 (1.105–2.245)^*^	3.046 (1.250–7.420)^*^
Heart failure	1.629 (1.402–1.894)^***^	1.377 (0.878–2.159)
Cardiac dysrhythmias	1.000 (0.870–1.150)	1.106 (0.737–1.660)
Anxiety	0.833 (0.720–0.964)^*^	0.796 (0.523–1.210)
CKD	1.560 (1.311–1.856)^***^	2.211 (1.387–3.525)^***^
Sleep disturbances	0.872 (0.753–1.010)	0.919 (0.601–1.404)
Anemia	1.592 (1.369–1.851)^***^	1.303 (0.825–2.058)

Abbreviation: *aOR* Adjusted odds ratio, *CI* Confidence interval, *CVD* Cardiovascular disease, *COPD* Chronic obstructive pulmonary disease, *CKD* Chronic kidney disease

Adiusted with gender, age, low income, other operation, comorbidity.*P < 0.05, **P < 0.01, ***P < 0.001

**Fig. 2:**
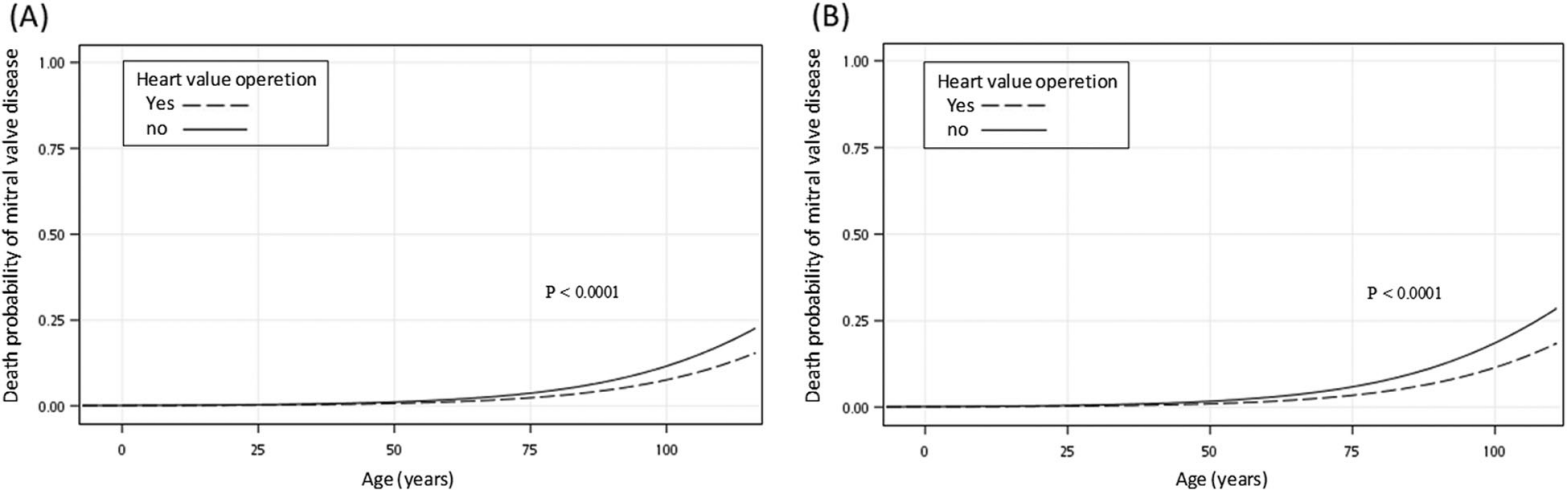
Logistic regression curve of factors associated with death in mitral valve disease

Fig. 2 presents the death probability associated with mitral valve disease in all age groups of all patients. Undergoing a heart valve operation for mitral valve disease was associated with a lower risk of death than not doing so in matched and non-matched patients. Figure 2A, the area under the receiver operating characteristic (ROC) curve is 0.8858. Figure 2B, the area under the ROC curve is 0.8504.

### Frequency of heart valve operation and other operations associated with death of patients with mitral valve disease

**Fig. 3:**
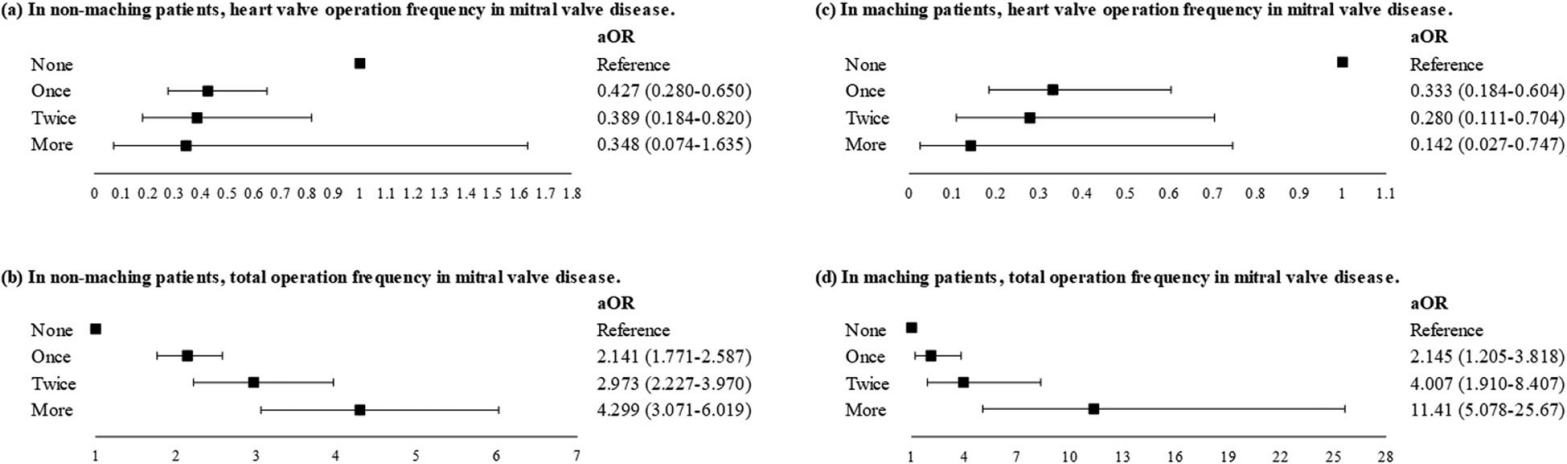
Risk of death associated with operation frequency in patients who underwent the heart valve operation, adjusted for sex, age, income, other operations, comorbidities, and operation frequency

Patients with mitral valve disease who underwent a heart valve operation once or twice exhibited a lower risk of death than other patients with mitral valve disease. However, patients who underwent a heart valve operation three or more times had a nonsignificantly lower risk of death from mitral valve disease (Fig. 3a). After matching, the patients with mitral valve disease who underwent the heart valve operation more times had the lower risk of mitral valve disease-related death (Fig. 3b). Patients with mitral valve disease who underwent more other operations had a higher risk of death than did other mitral valve disease patients (Fig. 3c, d).

## Discussion

This was the first national population-based study to evaluate the association between operations, including frequency and type thereof, and the mortality risk of mitral valve disease. We determined that patients with mitral valve disease had a higher rate of mortality than patients who underwent other operations, namely other CVD, respiratory condition, or urinary system operations. Patients who underwent a heart valve operation had a higher mortality rate among patients with comorbidity, namely diabetes, emphysema, and CKD. The mortality rate of patients with mitral valve disease who underwent the heart valve operations, including closed heart valvotomy, open heart valvuloplasty, replacement of heart valve, was lower than that of patients who did not. Finally, we purposed that patients with mitral valve disease who underwent more heart valve operations exhibited a lower rate of mortality, which contrasted with the higher mortality rate associated with other operations.

Prevention and early diagnosis of heart valve disease and prompt intervention could improve the long-term quality of life and mortality among patients [4, 8]. Patients suspected of heart failure were referred to the open access echocardiography service between 2001 and 2011 in the United Kingdom, and 37.5, 11.3, and 2.7% were diagnosed with mild, moderate, and severe heart valve disease, respectively. Moreover, evidence indicated that 13.5% of patients were diagnosed with mitral valve disease [9]. Mitral valve disease is the frequent cause of valvular heart disorders, including heart failure, and is associated with complications, namely arrhythmia, endocarditis, and sudden cardiac death. Mitral valve disease is a major cause of morbidity and mortality worldwide [2, 10]. The prevalence of mitral valve disease is more than 10% in adults older than 75 years [2]. The type of heart valve operations, which the procedure to repair diseased heart valves to treat heart valve diseases, include closed heart valvotomy, open heart valvuloplasty without replacement, and replacement of heart valve based on ICD-9-OP, ICD-9-CM. The mortality of mitral valve disease could be reduced by mitral balloon valvuloplasty which further improvement the lifespan and life quality for a long time [11]. The improvement effects of mitral valve repair in hospitalization, length of stay, and 10-year survival rate are better than mitral valve replacement in mitral valve disease patients which rule out older than 60 years of age or those requiring concomitant coronary artery bypass graft [12].The mortality rate of patients who underwent the first-ever open-heart mitral valve surgery had decreased in Finland from 1997 to 2014 despite the patients being older and having more comorbidities [13]. Greater volume of mitral procedures and mitral repair rates could decrease the mortality on elderly patients with mitral valve operations [14]. In previous study has purposed that the results for patients undergoing the redo heart valve operation have exhibited better than expected and improved outcomes [7]. At present, we further purposed that performing more time of heart valve operations, including closed heart valvotomy, open heart valvuloplasty, replacement of heart valve, could reduce the mortality rate of patients with mitral valve disease.

Other research contended that chronic lung disease, previous cardiac surgery, multiple valve repair, acute respiratory distress syndrome, cardiac arrest, and renal failure are the key risk factors associated with cardiovascular intensive care unit readmission after patients have underewnt heart valve operations [15]. The mobility of heart valve operations is increased by other operations, including respiratory condition operation [16], urinary system operations [17], and hemodialysis [18]. Here, we found that patients with mitral valve diseases who underwent other CVD system, respiratory condition, urinary system operations, and hemodialysis were more likely to have underwent a heart valve operation. Death risk assessment of other system operations was conducted using logistic regression analysis. Patients who underwent the other CVD system, respiratory condition, urinary system operation, and hemodialysis were 3.691, 3.210, 1.925, and 7.205 times, respectively, as likely to die from mitral valve disease more than controls. These results indicated that more time of hemodialysis, digestive system operations, respiratory condition operation, urinary tract operations, and other CVD system operations which excluded heart valve operations could increase the mortality rate of patients with mitral valve disease.

Patients with mitral valve disease who underwent a heart valve operation and had diabetes, emphysema, or CKD had 1.505, 2.623, and 3.760 times, respectively, the risk of death of controls. These findings were similar to those of another study that assessed patients to determine risk factors associated with cardiovascular intensive care unit readmission after a heart valve operation [15]. Diabetes was the key risk factor for mortality in patients with severe ischemic cardiomyopathy who underwent surgical mitral valve intervention [19]. Patients with CKD commonly also had heart valve disease and exhibited a high mortality rate when not treated with a heart valve operation. In addition, patients with serious CKD who underwent a heart valve operation had a relatively high rate of mortality [20]. Diabetes, emphysema, and renal impairment were other risk factors associated with heart valve disease [21]. This study was the first to demonstrate that patients who underwent a heart valve operation as well as another CVD, respiratory condition, or urinary system surgery or had diabetes, emphysema, or CKD exhibited the highest risk of death among patients with mitral valve disease.

### Limitations

Our study had several limitations. This study conducted analyses on the data in the NHIRD, and heart valve disease and heart valve operation diagnoses were strictly based on the ICD-9-CM, ICD-9-OP system. Patients with chronic rheumatic heart disease were identified by ICD-9-CM, but further characterized by the mitral and aortic valves cannot be classification. The source database did not include serious conditions and information regarding clinical parameters. In addition, the database also lacked details concerning heart valve operation type and duration. More, the prognosis, personal lifestyles and reasons, and living habits of reoperation patients could not be obtained from the NHIRD. Therefore, we could not assess surgical complications. Finally, the database was from Taiwan’s NHI system, and the population was located in Taiwan; these factors might limit the applicability of results to other countries.

## Conclusions

In conclusion, mitral valve disease is the most common heart valve disease, and the major treatment strategy is heart valve operations. The risk of death could be reduced by more frequently performing the heart valve operations in patients with mitral valve disease. The risk of death increases with a high frequency of other operations, containing other CVD system operations, respiratory conditions, urinary system operations, and hemodialysis. The risk of death increases with the critical comorbidities of mitral valve disease are diabetes, CKD, and emphysema. Present study could be good sufficient evidence to show the heart valve operations, including closed heart valvotomy, open heart valvuloplasty, and replacement of heart valve can lower the risk of death from valve disease. These conclusions could provide the important assistance benefit on clinical therapy for mitral valve disease patients.

## Data Availability

The data that support the findings of this study are available from NHIRD but restrictions apply to the availability of these data, which were used under license for the current study, and so are not publicly available. Data are however available from the authors upon reasonable request and with permission of NHIRD.

## Abbreviations

aOR: Adjusted odds ratios
CKD: Chronic kidney disease
CVD: Cardiovascular disease
ICD-9-CM: International Classification of Diseases, Ninth Revision, Clinical Modifications
LHID: Longitudinal Health Insurance Database
NHI: National Health Insurance
NHIRD: National Health Insurance Research Database
ORs: Odds ratios
